# Correlation between the etiology of severe hearing loss and endolymphatic hydrops

**DOI:** 10.1007/s00405-024-08993-3

**Published:** 2024-10-07

**Authors:** Sung-Min Park, Jin Hee Han, Jung Kyu Lee, Byung Se Choi, Yun Jung Bae, Byung Yoon Choi

**Affiliations:** 1 Department of Otorhinolaryngology-Head and Neck Surgery, College of Medicine, Kangnam Sacred Heart Hospital, Hallym University, Seoul, South Korea; 2 Department of Otorhinolaryngology-Head and Neck Surgery, Seoul National University Bundang Hospital, 300 Gumi-dong, Bundang-gu, Seongnam, 13620 Republic of Korea; 3 Sensory Organ Research Institute, Seoul National University Medical Research Center, Seoul, South Korea; 4 Department of Radiology, Seoul National University Bundang Hospital, 300 Gumi- dong, Bundang-gu, Seongnam, 13620 Republic of Korea

**Keywords:** Endolymphatic hydrops, Hearing loss, Magnetic resonance imaging, Mutation

## Abstract

**Purpose:**

This study aimed to investigate correlation between the presence of endolymphatic hydrops(EH) and factors such as causes of hearing loss, patient age, duration of deafness, and results of vestibular function tests.

**Methods:**

We retrospectively reviewed medical charts of 128 ears of cochlear implantees who were not considered relevant to Meniere’s disease.

**Results:**

When comparing group with genetic variants of *GJB2*,* SLC26A4*,* LMX1A* and other genetic mutation group, the proportion of vestibular EH and cochlear EH found in group with genetic variants of *GJB2*,* SLC26A4*,* LMX1A* was significantly higher than group with other genetic etiology (*p* < 0.01) or the group with all the other causes of hearing loss (*p* < 0.01). The rate of vestibular and cochlear EH detection was higher in younger patients (41.5% and 35.4%) than in older patients (25.4% and 20.6%). A higher ratio of vestibular and cochlear EH was observed in patients with a longer duration of deafness (37.5% and 31.3%) than those with a shorter duration of deafness (29.7% and 25.0%). The group with vestibular EH showed a higher incidence of abnormal findings in the caloric test (42.9%) than the group without vestibular EH (28.2%).

**Conclusion:**

Patients with genetic variants of *GJB2*,* SLC26A4*,* LMX1A*, younger patients, those with longer deaf durations showed a higher prevalence of vestibular and cochlear EH, implying EH appears to be formed as a developmental disorder in association with a certain set of genetic variants, rather than a phenotypic marker as a result of severe to profound hearing loss.

**Supplementary Information:**

The online version contains supplementary material available at 10.1007/s00405-024-08993-3.

## Introduction

Endolymphatic hydrops (EH) refers to a pathologic finding of distended endolymphatic space by enlargement of endolymphatic volume [1]. Primary EH is Meniere’s disease and more common. Secondary EH is acquired by various causes, including inflammatory or traumatic insult to the labyrinthine, and it is associated with episodic vertigo and fluctuating hearing loss [2]. However, exact mechanisms of formation of EH still remain unclear.

In the last decade, inner ear MRI became a crucial tool for detecting EH in vivo, in patients diagnosed with Meniere’s disease. In 2007, Nakashima et al. first found EH on MRI in living patients, based on the selective enhancement of the perilymphatic space 24 h after intratympanic administration of contrast media. Later, the same research team demonstrated that it was possible to assess the endolymphatic and perilymphatic spaces 4 h after the intravenous administration of contrast media. As an intratympanic injection is invasive, and there were no proven differences in the detection rate of EH between intratympanic and intravenous injections, the latter is more commonly used [3].

Although there have been studies on the genes involved in the formation of EH, no research has compared the prevalence of EH between groups with and without genetic variants related to hearing loss. To our knowledge, this study is the first to investigate the prevalence of EH in patients with severe to profound hearing loss. In this study, we hypothesized genetic factors are correlated with the formation of EH in patients without Meniere’s disease. Additionally, we explored the relationships between the presence of EH and factors such as patient age, duration of deafness, and results of vestibular function tests.

## Materials and methods

This was a retrospective observational study. The study protocol conformed to the guidelines of the Declaration of Helsinki and Korean Good Clinical Practice. This study was reviewed and approved by the Institutional Review Board of Seoul National University Bundang Hospital (IRB No. B-2309-852-104).

The medical charts of adult cochlear implantees in the Department of Otolaryngology at Seoul National University Bundang Hospital between June 2022 and July 2023 were retrospectively reviewed. HYDROPS MRI was performed on all patients aged 15 years and older who are scheduled to undergo CI surgery at our center. The inclusion criteria were the patients who underwent HYDROPS MRI before cochlear implantation (CI) and received CI surgery by a single surgeon (B.Y.C). Exclusion criteria were patients diagnosed with Meniere’s disease or those who were not suitable for HYDROPS MRI due to previous procedure such as CI.

### Genetic examination

Real-time PCR was performed using the U-TOP™ HL Genotyping Kit (SeaSun Biomaterials) with a CFX96 Real-Time PCR Detection system (Bio-Rad, Hercules, CA, USA) [4]. Eleven mutations in 5 sensorineural hearing loss genes were examined using this kit according to the manufacturer’s manual. The data were analyzed using Bio-Rad CFX manager v1.6 software (Bio-Rad). Mutations were characterized by the fluorescence signal of detection probes and corresponding melting temperatures. If the U-TOP screening test results were negative, a whole exome sequencing was performed [5, 6].

### Vestibular function test

The bithermal caloric test was performed preoperatively in all patients, and eye movements were recorded by a videonystagmography system. Each ear was irrigated alternately with a constant flow of air, with the temperature for warm or cool stimulation set at 50 and 24◦C, respectively. The duration of each caloric irrigation lasted 60 s. Upon each irrigation, the maximum slow phase velocity (SPVmax) of caloric nystagmus was measured. The percentage of canal paresis (CP%) was calculated using Jonkees formula, which was considered pathologic when it was 25% or more. If the summated SPVmax of the nystagmus was < 20°/s under four stimulation conditions, it was considered bilateral vestibular hypofunction [7].

All participants underwent vHIT (video head impulse test). We evaluated vHIT gains and gain asymmetry in all semicircular canal planes. Each vHIT gain was calculated from the ratio of the area under the curve (AUC) for the eye movement divided by the AUC for the head movement. Gain asymmetry (GA) was calculated from the gains obtained in response to rightward and leftward head impulses according to the following equation: GA = [(Gc - Gi)/(Gc + Gi)] X 100%, where Gc is the vHIT gain exciting the contralateral lateral canal and Gi is the vHIT gain exciting the ipsilateral lateral canal. We evaluated the corrective saccades (CS) incidence and the peak velocities and the interaural difference of the CS velocities (CSD) [8]. Pathologic CS was defined as when there were 2 or more CS incidents of a similar amplitude, with a CS peak velocity of ≥ 100°/s or a CSD ≥ 40°/s. When the vHIT gain was ≤ 0.7, it was evaluated as a decreased gain.

### HYDROPS MRI

Ears were evaluated by 3-Tesla MRI performed 4 h after intravenous injection of a standard dose (0.2 mL/kg body weight, i.e., 0.1 mmol/kg body weight) of gadodiamide hydrate (Gd). HYDROPS (hybrid of reversed image of positive endolymph signal and native image of positive perilymph signal) was used to evaluate the existence of endolymphatic hydrops (EH). Gd fills the perilymphatic space, but not the endolymphatic space, and these spaces can be identified as white and black areas, respectively. Thus, the presence of EH can be visualized as black areas surrounded by Gd-filled perilymph [9].

**Fig. 1:**
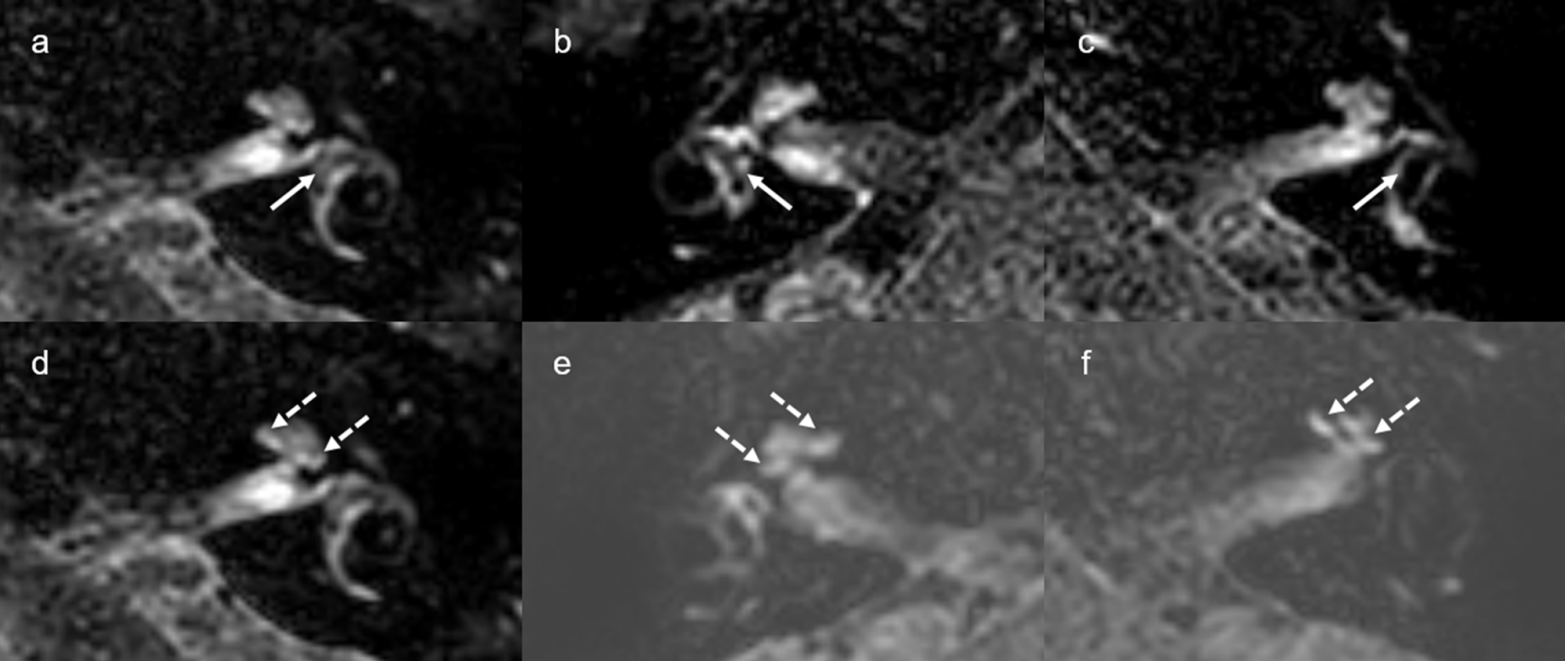
Delayed 3D FLAIR MRI of vestibular endolymphatic hydrops graded none (**a**), grade I (**b**), grade II (**c**), and cochlear endolymphatic hydrops graded none (**d**), grade I (**e**), grade II (**f**). Vestibular endolymphatic hydrops are pointed with white arrows, and cochlear endolymphatic hydrops are pointes with white dotted arrows

Two radiologist who did not know the patients’ clinical information classified the degree of EH in the vestibule and cochlea into three grades (none, grade I, and grade II) according to the criteria described by Barath et al. [10]. Grade I cochlear hydrops was characterized as mild dilation of the nonenhancing cochlear duct, sparing parts of the enhancing perilymph of the scala vestibuli. Grade I vestibular hydrops presented as distention of the endolymph space of the saccule or utricle or both, with the perilymphatic space still visible along the periphery of the bony vestibule. In grade II cochlear hydrops, the scala vestibuli was replaced by the maximally distended cochlear duct. In grade II vestibular hydrops, the bony vestibule was completely replaced by the dilated endolymphatic spaces (Fig. 1). In the interpretation of vestibular hydrops, the agreement rate between two radiologists was 85.2% (109/128), and in the interpretation of cochlear hydrops, the agreement rate was 96.1% (123/128). When there were discrepancies in the grading interpreted by two radiologists, consensus was reached through discussion.

### Statistical analysis

The data were analyzed using SPSS software (version 26.0 for Windows; SPSS, IBM, Armonk, NY, U.S.A.). The prevalence of EH was compared statistically among different groups using the chi-square test and Fisher exact test. Multiple regression analysis was performed to clarify the relationship between independent variables of age, genetic etiology, deaf duration, symptom of vertigo, abnormal vestibular function test and the presence of cochlear or vestibular EH. In addition, for sensitivity analysis, we conducted same logistic regression modeling with the outcome focused on grade II cochlear and vestibular EH. The level of significance was set at *p* < 0.05.

## Results

A total of 100 patients were included; 100 patients were implanted unilaterally, and 28 patients were implanted bilaterally. Among 100 patients, 21 patients had both vestibular and cochlear EH. All of the results of the whole exome sequencing are provided in supplementary Table 1. As a result of whole exome sequencing, a total of 13 variants was found in 28 patients (43 ears): *GJB2 variants* were found in six patients (10 ears), *SLC26A4 variants* in five patients (nine ears), *TMPRSS3 variants* in two patients (four ears), *CDH23 variants* in four patients (five ears), *MT-RNR1 variants* in two patients (two ears), an *OTOG variant* in one patient (two ears), a *LMX1A variant* in one patient (two ears), an *ILDR1 variant* in one patient (two ears), a *MT-TL1 variant* in one patient (two ears), an *ACTG1 variant* in one patient (one ear), a *SMPX variant* in one patient (one ear), a *POU3F4 variant* in one patient (one ear), a *MYH9 variant* in one patient (one ear).

Among 128 ears allegedly irrelevant to definite MD, 43 (33.6%) exhibited vestibular EH and 36 (28.12%) exhibited cochlear EH. Supplementary Table 2 depicts the grades of EH for each group when classified by the etiology of hearing loss. Fifty had an idiopathic cause, while the other 78 ears harbored a definite etiology. Among these 78 ears with a definite etiology, cases with a definite genetic etiology comprised 55.12% (*n* = 43), while sudden sensorineural hearing loss (*n* = 15) and chronic otitis media- related labyrinthitis (*n* = 13) were also the frequent etiology. In the idiopathic group, vestibular hydrops was observed in 40% (*n* = 20) and cochlear hydrops were observed in 28% (*n* = 14).

The genetic variants of *GJB2*,* SLC26A4*,* LMX1A* are involved in inner ear development and associated with the occurrence of asymmetric sensorineural hearing loss [11]. When comparing the group with genetic variants of *GJB2*,* SLC26A4*,* LMX1A* and other group with variants in *TMPRSS3*,* CDH23*,* ACTG1*,* MT-RNR1*,* SMPX*,* OTOG*,* MT-TL1*,* ILDR1*,* POU3F4*,* MYH9*, the proportion of vestibular EH found in the group with genetic variants of *GJB2*,* SLC26A4*,* LMX1A* (66.7%) was significantly higher than the group with other genetic causes (14.3%, *p* = 0.001). Similarly, the ratio of cochlear EH in the group with genetic variants of *GJB2*,* SLC26A4*,* LMX1A* (66.7%) was significantly higher than the group with other genetic causes (9.5%, *p* = 0.000, Table 1).

**Table 1:** Ratios of EH in GJB2, SLC26A4, LMX1A variants, other genetic variants, and all the other causes

Etiology of hearing loss	Vestibular hydrops (+)	Vestibular hydrops (-)	*P* value	Cochlear hydrops (+)	Cochlear hydrops (-)	*P* value
GJB2, SLC26A4, LMX1A variants	66.7 (14/21)	33.3 (7/21)	0.001	66.7 (14/21)	33.3 (7/21)	0.000
Other genetic variants	14.3 (3/21)	85.7 (18/21)		9.5 (2/21)	90.5 (19/21)	
GJB2, SLC26A4, LMX1A variants	66.7 (14/21)	33.3 (7/21)	0.001	66.7 (14/21)	33.3 (7/21)	0.000
All the other causes	27.1 (29/107)	72.9 (78/107)		20.6 (22/107)	79.4 (85/107)	

We also compared the ratios of EH in group with genetic variants of *GJB2*,* SLC26A4*,* LMX1A* and all the other causes of hearing loss, which comprises idiopathic, sudden sensorineural hearing loss, other genetic causes, otosclerosis, chronic otitis media related labyrinthitis, ANCA + vasculitis, postoperative labyringhitis, stroke- related, and cochlear nerve deficiency in Table 1. The proportion of vestibular EH found in group with genetic variants of GJB2, SLC26A4, LMX1A (66.7%) was significantly higher than group with all the other causes (27.1%, *p* = 0.001). The proportion of cochlear EH found in group with genetic variants of *GJB2*,* SLC26A4*,* LMX1A* (66.7%) was also significantly higher than group with all the other causes (20.6%, *p* = 0.000).

Table 2 analyzes the groups based on whether cochlear implantation (CI) was performed in individuals aged 55 and below versus those aged 55 and above, it was revealed that old age was not correlated with the detection rate of vestibular or cochlear EH during CI implementation (*p* = 0.053 and 0.064). Instead, the rate of vestibular and cochlear EH detection was higher in younger patients (41.5% and 35.4%) than in older patients (25.4% and 20.6%).

**Table 2:** Ratios of EH in young aged group (≤ 55 years), old aged group (> 55 years), short deafness duration group (≤ 10 years) and long deafness duration group (> 10 years) at implantation

Characteristics	Vestibular hydrops (-)	Vestibular hydrops (+)	*p* value	Cochlear hydrops (-)	Cochlear hydrops (+)	*P* value
Young age	38 (58.5%)	27 (41.5%)	0.053	42 (64.6%)	23 (35.4%)	0.064
Old age	47 (74.6%)	16 (25.4%)		50 (79.4%)	13 (20.6%)	
Short duration	45 (70.3%)	19 (29.7%)	0.349	48 (75.0%)	16 (25.0%)	0.618
Long duration	40 (62.5%)	24 (37.5%)		44(68.8%)	20 (31.3%)	

When patients were divided into those with a deaf duration of 10 years or less and those with more than 10 years of deafness duration, a higher ratio of vestibular EH observation was noted in patients with a longer duration of deafness (37.5%) than those with a shorter duration of deafness (29.7%). However, this difference was not statistically significant (*p* = 0.349). In addition, a higher ratio of cochlear EH was observed in long duration group (31.3%) than in short duration group (25.0%), but it was not significantly different (*p* = 0.618, Table 2).

Table 3 compares the proportions of abnormal findings in caloric test or vHIT results when dividing patients into groups with and without EH. The group with vestibular EH showed a higher incidence of abnormal findings in the caloric test (42.9%) than the group without vestibular EH (28.2%), although there was no statistically significant difference (*p* = 0.099). Meanwhile, in the group with cochlear EH, a higher incidence of abnormal vHIT gain was observed (32.4%) compared to the group without cochlear EH (18.5%), albeit no significant difference (*p* = 0.096). However, the presence of CS findings in vHIT tests was not related to cochlear or vestibular EH.

**Table 3:** Correlation between vestibular function loss and presence of EH in MRI

Incidence of abnormal findings based on	Vestibular hydrops (+)	Vestibular hydrops (-)	*P* value	Cochlear hydrops (+)	Cochlear hydrops (-)	*P* value
caloric test, % (No./total No.)	42.9 (18/42)	28.2 (24/85)	0.099	34.3 (12/35)	32.6 (30/92)	0.858
vHIT gain, % (No./total No.)	21.4 (9/42)	22.6 (19/84)	0.880	32.4 (11/34)	18.5 (17/92)	0.096
vHIT CS, % (No./total No.)	11.9 (5/42)	17.9 (15/84)	0.389	14.7 (5/34)	16.3 (15/92)	0.827
caloric test or vHIT, % (No./total No.)	28.6 (12/42)	27.4 (23/84)	0.888	38.2 (13/34)	23.9 (22/92)	0.111

vHIT, video head impulse test; CS, corrective saccade;

## Discussion

The most common cause of severe to profound haring loss in our study population was idiopathic, which constitutes 39.1% (50/128). This result significantly aligns with a previous meta-analysis study investigating the causes of severe to profound hearing loss in cochlear implanted children, which states that 40.3% of them had ‘unknown’ etiology [12]. Non-syndromic etiology constitutes 20.3%(26/128) among our study population, which is not different from previous meta-analysis, which shows 22.4% had ‘Non-syndromic’ etiology [12]. It was observed that the prevalence of etiologies of hearing loss in cochlear implanted adults and children is not significantly different from the prevalence observed in studies which include only pediatric patients.

In our study, there was high prevalence of endolymphatic hydrops in patients with genetic mutations of *GJB2*,* SLC26A4*, and *LMX1A*. In ears with *GJB2 variants*, 37.5% (3/8) had vestibular hydrops and 36.3% (4/7) had cochlear hydrops in our cohort. In ears with *SLC26A4 variants*, all of them had vestibular hydrops and 88.8% (8/9) had cochlear hydrops. This result is consistent with previous research suggesting that majority of patients with *SLC26A4 mutations* have EH in vestibule (8/10 ears) and cochlear (7/10 ears) [13]. It can be assumed that human mutations in *SLC26A4* have defect in pendrin expression, which is required for embryonic development of the inner ear. Therefore, EH might develop embryonically in these patients [13]. Moreover, all of two patients with *LMX1A variants* had both vestibular and cochlear hydrops in our cohort. *LMX1A* is essential in development of nonsensory epithelia of the ear [14]. As the prevalence of EH is high in those with genetic mutations that affect inner ear development, it can be speculated that a certain set of genes plays a crucial role in the formation of EH. Coincidentally, asymmetric sensorineural hearing loss is commonly found in *GJB2*,* SLC26A4*, and *LMX1A variants* [15–17]. Several other genetic mutations were identified to cause EH, such as mutations in *auaporins 1–4*, variants of voltage gated potassium channels, KCNQ1 and KCNE1 and variant of human alpha-adducin gene (*ADD1*), Salt-Inducible Kinase-1 gene (*SIK1*), and Solute Carrier Family Member 1 (*SLC8A1*) gene [18]. Among 28 patients who had CI surgery on both ears, the concordance rate for the presence of vestibular hydrops between both ears was 82.1%(23/28). Similarly, the concordance rate for the presence of cochlear hydrops between both ears was also 82.1% (23/28). This high concordance rated supports the evidence that genetic factors my influence the formation of endolymphatic hydrops.

In patients with idiopathic hearing loss and sudden sensorineural hearing loss, although not as high as patients with genetic variations of *GJB2*,* SCL26A4*, and *LMX1A*, the ratios of EH detected in HYDROPS MRI was relatively high, reaching 20–40%. Therefore, in patients with severe hearing loss (threshold above 70dB) due to idiopathic or sudden sensorineural hearing loss, EH seems to play a significant role as a cause or as a result of a severe hearing loss. Since EH is not prevalent in every group in the supplementary Table 2, it can be said EH is more likely to be caused by the developmental disorder in association with a certain set of genetic variants [19], rather than a phenotypic marker as a result of severe to hearing loss.

The detection rate of vestibular or cochlear EH was higher in younger patients according to our study. Several studies supported that asymptomatic EH and young age is strongly related [20]. In one study analyzed 118 temporal bones in individuals aged 10 or younger, Reissner’s membrane was bulged in 54.2% [21]. Due to the development of MD typically occurring in middle age, along with very low incidence of meniere’s disease in younger people, implies that EH is a cause rather than a consequence of meniere’s disease [20]. However, in a study involving patients with normal vestibulocochlear tests, it was suggested that age is positively correlated with volume of endolymphatic space [22]. This implies that endolymphatic space may increase with age within normal range, but when it reached a size considered to be EH, it does not correlate with age according to our results. If there is a higher prevalence of EH in the young age group, it implies that EH is a significant factor in the mechanism of hearing loss. Conversely, if there is a higher prevalence in the old age group, it suggests that EH occurs as a result of severe hearing loss. Therefore, our study result supports that EH is formed by developmental disorder associated with a certain set of genetic variants, rather than a phenotypic marker as a consequence of severe to profound hearing loss.

Delayed endolymphatic hydrops is a condition which occurs in patients who have a profound hearing loss in one ear and develop episodic vertigo after a prolonged period of time [23]. It is likely that in patients with profound hearing loss, delayed endolymphatic hydrops developed, leading to a higher detection rate of endolymphatic hydrops in a longer duration of deafness group in our cohort. A study with sudden deafness who had abnormal VEMP responses but normal caloric function also reported that these people may subsequently develop secondary hydrops with a mean interval of eight year [24].

Several studies have conducted vestibular evoked myogenic potential (VEMP) to analyze correlations between EH with saccule. Young et al. [25] reported the absence of VEMP was not related to vestibular hydrops on MRI in patients with define Meniere’s disease. Meanwhile, Katayama et al. [26] revealed a significant relationship between EH and VEMP, with vestibular hydrops having stronger effect than cochlear hydrops. To the best of our knowledge, this was the first study which examined the correlation between the presence of endolymphatic hydrops confirmed by HYDROPS MRI and the results of vestibular function test in severe hearing loss patients without Meniere’s disease. In this study, the group with vestibular EH showed a higher incidence of abnormal findings in the caloric test than the group without vestibular EH, but the higher incidence of abnormal results of vHIT was found in the group without vestibular EH. This result coincides with previous study, which reported abnormal caloric response in the presence of a preserved vHIT was common in patients with delayed endolymphatic hydrops [27]. This implies the presence of delayed EH in HYDROPS imaging might be associated with abnormal finding in caloric tests.

## Conclusion

We investigated the prevalence and correlates of EH in 128 ears not considered relevant to definite Ménière’s disease (MD). Among these ears, 33.6% exhibited vestibular EH and 28.12% exhibited cochlear EH. Etiologies of hearing loss varied, with 50 cases being idiopathic and 78 having a definite etiology, including genetic factors. Patients with genetic variants of *GJB2*,* SLC26A4*, and *LMX1A* showed significantly higher detection of EH compared to other causes of hearing loss. Younger implantees and those with longer deaf durations tended to have higher EH detection rates. EH is correlated with developmental disorders with a certain set of genetic variants, rather than merely being a phenotypic marker as a result from severe to profound hearing loss. The presence of vestibular EH also correlated with abnormal findings in caloric tests.

## Electronic supplementary material

Below is the link to the electronic supplementary material.

Supplementary Material 1

## Data Availability

The datasets used and/or analysed during the current study available from the corresponding author on reasonable request.
