# Supplementary material for: Correlation between the etiology of severe hearing loss and endolymphatic hydrops
Source: Eur Arch Otorhinolaryngol. 2024 Oct 7;282(2):781–7. doi: 10.1007/s00405-024-08993-3 (PMC11805888; doi:10.1007/s00405-024-08993-3)
Supplement: Supplementary file 1 — Supplementary Material 1 [file 405_2024_8993_MOESM1_ESM.docx]

**Title: Correlation between the Etiology of Severe Hearing Loss and Cochlear Hydrops**

**European Archieves of Oto-Rhino-Laryngology**

**Sung-Min Park^a^, Jin Hee Han^b,c^, Byung Se Choi^d^, Yun Jung Bae^d^, Byung Yoon Choi^b,c^**

^a^Department of Otorhinolaryngology-Head and Neck Surgery, College of Medicine, Kangnam Sacred Heart Hospital, Hallym University,Seoul, South Korea;

^b^Department of Otorhinolaryngology-Head and Neck Surgery, Seoul National University Bundang Hospital, Seongnam, South Korea;

^c^Sensory Organ Research Institute, Seoul National University Medical Research Center, Seoul, South Korea;

*^d^Department of Radiology, Seoul National University Bundang Hospital, Seongnam, South Korea*

Corresponding Coauthor:

1. Byung Yoon Choi, MD, PhD, Department of Otorhinolaryngology–Head & Neck Surgery, Seoul National University Bundang Hospital, 300 Gumi-dong, Bundang-gu, Seongnam 13620, Republic of Korea.

Tel: +82-31-787-7406, Fax: +82-31-787-4057, E-mail: [choiby2010@gmail.com](mailto:choiby2010@gmail.com)

2. Yun Jung Bae, MD, PhD, Department of Radiology, Seoul National University Bundang Hospital, 300 Gumi-dong, Bundang-gu, Seongnam 13620, Republic of Korea.

Tel: +82-31-787-7635, Fax: +82-31-787-4011, E-mail: [bae729@gmail.com](mailto:bae729@gmail.com)

Supplementary table 1. Whole genome sequencing results of 21 patients with genetic cause of hearing loss

| **Patient ID** | **Gender/ Age** | **Phenotype** | **Gene** | **NCBI reference sequence** | **HGVS** | **Zygosity** | **Insilico Predictions** | | | **Alternative Allele Frequency** | **Clinical Significance**  **(ClinVar)** |
| --- | --- | --- | --- | --- | --- | --- | --- | --- | --- | --- | --- |
|  |  |  |  |  |  |  | **CADD Phred** | **REVEL** | **SpliceAI**  **(Impact)** |  |  |
| SB1172-1904 | M/35 | Both postlingual progressive profound deafness | TMPRSS3 | NM_001256317.3 | c.783-1G>A | Heterozygote | 33.00 | NA | 0.99  (Acceptor Loss) | T=0.000008  (2/248818, GnomAD_exome)  T=0.0003  (1/2922, KOREAN) | Pathogenic/Likely pathogenic |
|  |  |  |  |  | c.391G>A:p.Asp131Asn | Heterozygote | 26.30 | 0.339 | NA | T=0.000093  (13/140256, GnomAD)  T=0.0007  (2/2922, KOREAN) | Uncertain significance |
| SB1030-1699 | F/60 | Both downsloping SNHL | TMPRSS3 | NM_001256317.3 | c.916G>A:p.Ala306Thr | Heterozygote | 26.50 | 0.851 | NA | T=0.000193  (27/140222, GnomAD)  T=0.0014  (4/2922, KOREAN) | Pathogenic/Likely pathogenic |
|  |  |  |  |  | c.1192G>A:p.Gly398Arg | Heterozygote | 32.00 | 0.979 | NA | T=0.000007  (1/140186, GnomAD) | Not Reported |
| SB839-1786 | F/58 | Severe to profound deafness | GJB2 | NM_004004.6 | c.427C>T:p.Arg143Trp | Heterozygote | 29.30 | 0.918 | NA | A=0.000207  (29/140250, GnomAD)  A=0.0007  (2/2922, KOREAN) | Pathogenic |
|  |  |  |  |  | c.235del:p.Leu79Cys*fs**3 | Heterozygote | 32.00 | NA | NA | delG=0.000214  (30/140258, GnomAD) | Pathogenic |
| SB1314-2075 | F/77 | Asymmetric borderline SNHL | GJB2 | NM_004004.6 | c.583A>G:p.Met195Val | Heterozygote | 25.50 | 0.962 | NA | C=0.000007  (1/140246, GnomAD)  C=0.0014  (4/2922, KOREAN) | Pathogenic |
| SB1315-2076 | M/51 | Both severe to profound SNHL | GJB2 | NM_004004.6 | c.109G>A:p.Val37Ile | Heterozygote | 21.70 | 0.656 | NA | T=0.003537  (496/140244, GnomAD)  T=0.0068  (20/2922, KOREAN) | Pathogenic |
| SB1316-2077 | F/69 | Deafness | GJB2 | NM_004004.6 | c.109G>A:p.Val37Ile | Heterozygote | 21.70 | 0.656 | NA | T=0.003537  (496/140244, GnomAD)  T=0.0068  (20/2922, KOREAN) | Pathogenic |
| SB1317-2078 | M/70 | Both progressive profound SNHL | GJB2 | NM_004004.6 | c.23C>T:p.Thr8Met | Heterozygote | 16.46 | 0.632 | NA | A=0.000064  (9/140242, GnomAD)  A=0.0014  (4/2922, KOREAN) | Uncertain significance |
|  |  |  |  |  | c.235del:p.Leu79Cys*fs**3 | Heterozygote | 32.00 | NA | NA | delG=0.000214  (30/140258, GnomAD) | Pathogenic |
| SB1161-1887 | F/31 | Both prelingual onset profound deafness | GJB2 | NM_004004.6 | c.427C>T:p.Arg143Trp | Heterozygote | 29.30 | 0.918 | NA | A=0.000207  (29/140250, GnomAD)  A=0.0007  (2/2922, KOREAN) | Pathogenic |
|  |  |  |  |  | c.235del:p.Leu79Cys*fs**3 | Heterozygote | 32.00 | NA | NA | delG=0.000214  (30/140258, GnomAD) | Pathogenic |
| SB1049-1719 | F/35 | Both deafness,  ski-slope type HL | CDH23 | NM_022124.6 | c.3076del:p.Leu1026Ser*fs**27 | Heterozygote | absent | NA | NA | absent | Pathogenic |
|  |  |  |  |  | c.4762C>T:p.Arg1588Trp | Heterozygote | 24.20 | 0.421 | NA | T=0.0008 (4/5008, 1000G)  T=0.0010 (3/2922, KOREAN) | Likely pathogenic |
| SB1319-2081 | F/50 | Lt intractable tinnitus under Both asymmetrical downsloping SNHL (Rt<Lt) | CDH23 | NM_022124.6 | c.6713-1G>A | Heterozygote | 34.00 | NA | 0.99  (Acceptor Loss) | absent | Not Reported |
|  |  |  |  |  | c.5986G>A:p.Ala1996Thr | Heterozygote | 25.90 | 0.507 | NA | A=0.000029  (4/140198, GnomAD) | Not Reported |
| SB1027-1696 | F/30 | Both deafness | CDH23 | NM_022124.6 | c.6253+1G>A | Heterozygote | 34.00 | NA | 1.00  (Donor Loss) | absent | Likely pathogenic |
|  |  |  |  |  | c.553G>A:p.Gly185Ser | Heterozygote | 24.30 | 0.890 | NA | A=0.000007  (1/140232, GnomAD)  A=0.0003  (1/2922, KOREAN) | Not Reported |
| SB1081-1755 | F/64 | Deafness | ACTG1 | NM_001614.5 | c.151G>A:p.Asp51Asn | Heterozygote | 25.70 | 0.842 | NA | absent | Not Reported |
| SB1318-2079 | M/54 | Both downsloping hearing loss (severe to profound Rt<Lt) | MT-RNR1 | NC_012920.1 | m.1555A>G | Homoplasmy | NA | NA | NA | G=0.0007 (2/2922, KOREAN) | Pathogenic/ Drug response |
| SB1082-1757 | F/40 | Enlarged vestibular aqueduct syndrome | SLC26A4 | NM_000441.2 | c.412G>C:p.Val138Leu | Heterozygote | 25.20 | 0.823 | NA | C=0.0003 (1/2922, KOREAN) | Pathogenic/Likely pathogenic |
|  |  |  |  |  | c.1149+3A>G | Heterozygote | 23.70 | NA | 0.48  (Donor Loss) | G=0.000024  (6/251110, GnomAD_exome)  G=0.0007  (2/2922, KOREAN) | Pathogenic |
| SB1129-1843 | F/18 | Enlarged vestibular aqueduct syndrome | SLC26A4 | NM_000441.2 | c.2168A>G:p.His723Arg | Homozygote | 25.20 | 0.933 | NA | G=0.000036  (5/140300, GnomAD)  G=0.0065  (19/2922, KOREAN) | Pathogenic/Likely pathogenic |
| SB1174-1906 | M/19 | Enlarged vestibular aqueduct syndrome | SLC26A4 | NM_000441.2 | c.2168A>G:p.His723Arg | Homozygote | 25.20 | 0.933 | NA | G=0.000036  (5/140300, GnomAD)  G=0.0065  (19/2922, KOREAN) | Pathogenic/Likely pathogenic |
| SB1196-1929 | M/27 | Enlarged vestibular aqueduct syndrome | SLC26A4 | NM_000441.2 | c.2168A>G:p.His723Arg | Heterozygote | 25.20 | 0.933 | NA | G=0.000036  (5/140300, GnomAD)  G=0.0065  (19/2922, KOREAN) | Pathogenic/Likely pathogenic |
|  |  |  |  |  | c.919-2A>G | Heterozygote | 34.00 | NA | 1.00  (Acceptor Loss) | G=0.000178  (25/140276, GnomAD)  G=0.0010  (3/2922, KOREAN) | Pathogenic |
| SB1321-2083 | F/15 | Enlarged vestibular aqueduct syndrome | SLC26A4 | NM_000441.2 | c.2168A>G:p.His723Arg | Heterozygote | 25.20 | 0.933 | NA | G=0.000036  (5/140300, GnomAD)  G=0.0065  (19/2922, KOREAN) | Pathogenic/Likely pathogenic |
| SH310-701 | M/18 | Asymmetrical SNHL | SMPX | NM_014332.2 | c.231A>C:p.Lys77Asn | Hemizygote | 25.60 | 0.298 | NA | absent | Not Reported |
| SB1117-1831 | F/54 | Both downsloping SNHL (profound) | OTOG | NM_001277269.1 | c.3043-2A>G | Heterozygote | 33.00 | NA | 0.94  (Acceptor Loss) | absent | Not Reported |
|  |  |  |  |  | c.6131C>T:p.Thr2044Ile | Heterozygote | 2.90 | 0.007 | NA | absent | Not Reported |
| SB1199-1932 | M/60 | Progressive cochleo vestibular dysfunction | MT-RNR1 | NC_012920.1 | m.1555A>G | Homoplasmy | NA | NA | NA | G=0.0007  (2/2922, KOREAN) | Pathogenic/ Drug response |
| SB1120-1834 | M/51 | MELAS, Both progressive postlingual profound deafness | MT-TL1 | NC_012920.1 | m.3243A>G | Heteroplasmy | NA | NA | NA | absent | Pathogenic/Likely pathogenic |
| SB1164-1893 | M/18 | Both perilingual SNHL | LMX1A | NM_001174069.1 | c.743C>T:p.Ala248Val | Heterozygote | 27.40 | 0.872 | NA | A=0.000007  (1/140220, GnomAD) | Not Reported |
| SB1158-1881 | F/51 | Both progresive SNHL (severe to profound) | ILDR1 | NM_001199799.1 | c.358C>T:p.Arg120Cys | Heterozygote | 32.00 | 0.736 | NA | A=0.000000  (0/138150, GnomAD)  A=0.0003  (1/2914, KOREAN) | Not Reported |
|  |  |  |  |  | c.206C>A:p.Pro69His | Heterozygote | 26.80 | 0.792 | NA | T=0.000014  (2/140172, GnomAD) | Likely pathogenic |
| SB606-1179 | M/34 | Both IP type III | POU3F4 | NM_000307.4 | Genomic inversion upstream region of POU3F4 | Hemizygote | NA | NA | NA | absent | Not Reported |
| SB1170-1902 | F/55 | Both Ski Slope SNHL | MYH9 | NM_002473.4 | c.2163T>G:p.Tyr721* | Heterozygote | 33.00 | NA | NA | absent | Not Reported |
| SB1320-2082 | F/71 | Both severe SNHL | CDH23 | NM_022124.6 | c.719C>T:p.Pro240Leu | Heterozygote | 24.60 | 0.516 | NA | T=0.000007  (1/140284, GnomAD)  T=0.0014 (4/2922, KOREAN) | Pathogenic |

SNHL, Sensorineural hearing loss; NA, Not applicable; IP type III, Cochlear incomplete partition type III; HGVS: Human Genome Variation Society (<https://www.hgvs.org/>); Sequence Variant Nomenclature (<https://mutalyzer.nl/>); CADD: Combined Annotation Dependent Depletion (<https://cadd.gs.washington.edu/>); REVEL: Rare Exome Variant Ensemble Learner (<https://sites.google.com/site/revelgenomics/>); KOVA: Korean Variant Archive for a reference database of genetic variations in the Korean population (<https://www.kobic.re.kr/kova/>); gnomAD: The Genome Aggregation Database (<https://gnomad.broadinstitute.org/>); spliceAI : <https://github.com/Illumina/SpliceAI>; ClinVar : <https://www.ncbi.nlm.nih.gov/clinvar>

Supplementary table 2. Grade of EH according to the etiology of hearing loss

| Etiology | Vestibular hydrops | | | Cochlear hydrops | | |
| --- | --- | --- | --- | --- | --- | --- |
|  | none | grade I | grade II | none | grade I | grade II |
| *TMPRSS3 variant* | 3 | 1 | 0 | 4 | 0 | 0 |
| SSNHL | 12 | 3 | 0 | 12 | 3 | 0 |
| *GJB2 variant* | 7 | 2 | 1 | 6 | 3 | 1 |
| Idiopathic | 31 | 17 | 3 | 37 | 10 | 4 |
| *CDH23 variant* | 5 | 0 | 0 | 5 | 0 | 0 |
| Otosclerosis | 0 | 1 | 0 | 1 | 0 | 0 |
| COM related labyrinthitis | 12 | 1 | 0 | 11 | 1 | 1 |
| *ACTG1 variant* | 1 | 0 | 0 | 0 | 1 | 0 |
| *MT-RNR1 variant* | 2 | 0 | 0 | 2 | 0 | 0 |
| *SLC26A4 variant* | 0 | 8 | 1 | 1 | 5 | 3 |
| ANCA+vasculitis | 1 | 0 | 0 | 1 | 0 | 0 |
| Stroke | 1 | 0 | 0 | 1 | 0 | 0 |
| *SMPX variant* | 0 | 1 | 0 | 1 | 0 | 0 |
| *OTOG variant* | 2 | 0 | 0 | 1 | 1 | 0 |
| *MT-TL1 variant* | 2 | 0 | 0 | 2 | 0 | 0 |
| CND | 1 | 1 | 0 | 1 | 1 | 0 |
| *Lmx1a variant* | 0 | 1 | 1 | 0 | 1 | 1 |
| Postoperative labyrinthitis | 2 | 0 | 0 | 2 | 0 | 0 |
| *ILDR1 variant* | 2 | 0 | 0 | 2 | 0 | 0 |
| *POU3F4 variant* | 1 | 0 | 0 | 1 | 0 | 0 |
| *MYH9 variant* | 0 | 1 | 0 | 1 | 0 | 0 |

EH, endolymphatic hydrops; SSNHL, sudden sensorineural hearing loss; COM, chronic otitis media; CND, cochlear nerve deficiency.
